# Engaging Tomorrow’s Doctors in Clinical Ethics: Implications for Healthcare Organisations

**DOI:** 10.1007/s10728-020-00403-z

**Published:** 2020-09-07

**Authors:** Laura L. Machin, Robin D. Proctor

**Affiliations:** 1 Lancaster Medical School, Faculty of Health and Medicine, Lancaster University, Lancaster, UK; 2 University Hospitals of Morecambe Bay NHS Foundation Trust, Lancaster, UK

**Keywords:** Clinical ethics, Ethical practices, Ethical awareness, Ethical sensitivity, Healthcare organisations, Medical students

## Abstract

Clinical ethics can be viewed as a practical discipline that provides a structured approach to assist healthcare practitioners in identifying, analysing and resolving ethical issues that arise in practice. Clinical ethics can therefore promote ethically sound clinical and organisational practices and decision-making, thereby contributing to health organisation and system quality improvement. In order to develop students’ decision-making skills, as well as prepare them for practice, we decided to introduce a clinical ethics strand within an undergraduate medical curriculum. We designed a programme of clinical ethics activities for teaching and assessment purposes that involved using ethical frameworks to analyse hypothetical and real-life cases in uni- and inter- professional groups. In this paper, we draw on medical student feedback collected over 6 years to illustrate the appeal to students of learning clinical ethics. We also outline the range of benefits for students, healthcare organisations, and the field of clinical ethics arising from tomorrow’s doctors experiencing clinical ethics early in their training. We conclude by briefly reflecting on how including clinical ethics within tomorrow’s doctors curricular can secure and continue future engagement in clinical ethics support services in the UK, alongside the dangers of preparing students for organisational cultures that might not (yet) exist. We anticipate the findings presented in the paper will contribute to wider debates examining the impact of ethics teaching, and its ability to inform future doctors’ practice.

## Introduction

Clinical ethics can be viewed as a practical discipline that provides a structured approach to assist healthcare practitioners in identifying, analysing and resolving ethical issues that arise in practice [35]. In practice, clinical ethics typically involves the provision of ethics expertise into clinical education, policy development and the care of individual patients. Clinical ethics can therefore promote ethically sound clinical and organisational practices and decision-making, thereby contributing to health organisation and system quality improvement [11, 24]. In order to develop students’ decision-making skills, as well as prepare them for practice, we decided to introduce a clinical ethics strand within an undergraduate medical curriculum. We designed a programme of clinical ethics activities for teaching and assessment purposes that involved using ethical frameworks to analyse hypothetical and real-life cases in uni- and inter- professional groups.

In this paper, we draw on medical student feedback collected over 6 years to illustrate the appeal to students of learning clinical ethics. We also outline the range of benefits for students, healthcare organisations, and the field of clinical ethics arising from tomorrow’s doctors experiencing clinical ethics early in their training. In particular, students reported their decision-making skills developed through the clinical ethics activities, and students perceived their ethical sensitivity and ethical awareness evolved when on clinical placement. We conclude by briefly reflecting on how including clinical ethics within tomorrow’s doctors curricular can secure and continue future engagement in clinical ethics support services in the UK, alongside the dangers of preparing students for organisational cultures that might not (yet) exist. We anticipate the findings presented in the paper will contribute to wider debates examining the impact of ethics teaching, and its ability to inform future doctors’ practice.

## The State of Clinical Ethics in the UK

In the late 1970s, clinical ethics emerged as an applied field of biomedical ethics that focuses on individual cases within clinical practice and the practical consequences, such as the moral and ethical issues that arise out of the clinician-patient/family relationship [42, 43, 45]. Clinical ethics has been described as “a discipline that aims to make ethical decisions more orderly, systematic and rational” and therefore “deals with concrete judgements in situations in which action must be taken despite uncertainty” [35].

In the UK, there have been high-profile champions for clinical ethics, particularly surrounding making Clinical Ethics Committees (CECs) and education available to healthcare practitioners. A report from the Royal College of Physicians (RCP) [38] concluded ethics support is required wherever healthcare is provided, as the practice of ethical medicine depends on the ethical understanding by all healthcare professionals. For the RCP, both clinical ethics support and education are equally needed, whilst the Nuffield Council on Bioethics [33] has argued that those working in neonatal intensive care units are in particular need of rapid access to CECs. The Care Quality Commission has also recognised the important role of CECs in their reports when visiting children’s hospitals where such Committees exist (Machin & Wilkinson, accepted). More recently, the UK Clinical Ethics Network (UKCEN) [46] claimed that when viewing the NHS as a moral body, where staff are part of a moral community and make moral decisions on a daily basis, CECs are necessities for healthcare organisations to fulfil its duty of care to their staff and patients.

Despite the on-going vocal support for clinical ethics, on the ground there has been little financial investment or regulatory support underpinning its presence in the UK, which is disturbing when contemplating the state of ethics training available to healthcare practitioners. It is well known that current senior doctors have not received such in-depth ethical training as deemed essential by the General Medical Council for today’s medical students [9]. Research has also highlighted the lack of ethical and legal training available once doctors qualify [15, 23]. Healthcare practitioners therefore could benefit from having access to CECs that aim to support doctors, hospital management, patients and relatives when confronted with an ethical concern, question or dilemma [29]. Yet it appears the number of CECs available are dwindling, as there is a reported decrease in CECs registered with the UKCEN [2], although it is important to note that it is not compulsory for CECs to register with UKCEN in order for them to operate. Moreover, it is not a legal requirement for NHS hospitals to have clinical ethics support available [24] meaning CECs and clinical ethicists within hospitals are optional in the UK and therefore not every hospital has access to a CEC or a clinical ethicist. Clinical ethics in the UK therefore tends to function as a voluntary system, “…without significant institutional support or resources” [1, 46] as staff who form the CECs receive no financial benefit, and rarely are their workloads reduced in order to accommodate the additional work of the CECs. At a time when the present social and political conditions may mean that healthcare workers are in danger of losing their capacity for moral sensitivity [30, 47], the training of medical students and qualified doctors in clinical ethics so they can develop their awareness, skills and attitudes feels more urgent and essential than ever [38].

## The Value of Clinical Ethics Education

Researchers have asked if knowledge of ethics translate into action where and when it is considered to really matter, such as on the wards, in the community or with clients [5, 27, 44]. Others have questioned if ethical behaviour can be taught and learnt [17] therefore raising doubts about the status and place of ethics teaching [28]. For some staff and students, the teaching of ethics can be perceived as “a scaled down version of teaching moral philosophy to philosophy students” [8] making it appear too abstract or removed from practice [18], and leaving some students struggling to see the value or relevance of the topic [6].

Introducing clinical ethics to medical students can help to address some of these issues relating to the teaching of ethics, such as student engagement with the topic, and highlighting the relevance of the topic to everyday healthcare practice. Clinical ethics can encourage students to combine their knowledge of ethical theory with real-life ethical problems that arise in clinical settings [1]. Students are therefore required to apply their knowledge, which implicitly reinforces the relevance of ethics learning to them [20].

The practical focus of clinical ethics also means that students can hone their decision-making skills [1]. Students learn the difference between technical facts, personal opinion, and professional values, as well as appreciate reasoned argument and justifiable clinical ethical decision-making [32]. In essence, clinical ethics education complements medical students’ wider professional development and helps them establish the foundational behaviours of professional practice [12]. This is particularly pertinent when contemplating the potential for ‘moral distress’ [13] and ‘ethical erosion’ [44]. Students may feel pressured to relinquish their ethical values whilst on placements as they observe ‘unethical’ behaviours from qualified practitioners [36]. In turn such experiences can formulate an alternative, ‘hidden curriculum’, to that of the one proposed by the medical school, which should be adhered to in order to qualify [16].

For those who teach clinical ethics, they report it challenges the ‘moral distress’ and ‘ethical erosion’ as it focuses upon developing students’ capabilities to identify and manage ethical dilemmas whilst on placements [20]. It sharpens students’ awareness of the ethical issues in everyday clinical work [10]. Clinical ethics activities can therefore provide reflective space and time for students to explore any ethical conflicts they have witnessed during placements, as well as the opportunity to examine staff behavior in the clinical setting [10]. Innovative medical educationalists, wanting to demonstrate clinical ethics in practice, have initiated pseudo CECs for medical students. For these students, the pseudo CECs played a useful role in offering advice, support and information, and were a useful experience for those wishing to learn about clinical ethical decision-making and hospital ethics committees [20, 37].

However, whilst educators are responsible for imparting knowledge and fostering learning to ensure that tomorrow’s practitioners have the skills to recognize the moral aspects of their practice, the organisation is also considered to play a role [19, Talash et al., accepted]. Arguably, healthcare administrators create conditions in the workplace that can either facilitate or prohibit an employee from making use of this training. Any recommendations and interventions proposed therefore have implications for both educators and healthcare administrators [19]. Focusing on the organisation in this way allows us to cultivate an ethos supportive of the practice changes needed so that the presence of clinical ethics is accepted, acknowledged as needed, and in turn has the potential to shape the organisation itself [42].

## Introducing Clinical Ethics into a Medical Curriculum

At Lancaster Medical School, the five-year medical undergraduate curriculum is predominately delivered through problem-based learning and is supported with lectures and recommended resources. The curriculum consists of four themes, including Professional Practice, Values and Ethics (PPVE), whereby the students learn about medical ethics, law and professionalism throughout the programme. A range of teaching methods are utilised when delivering PPVE including workshops, debates, and interprofessional education (see [22]). Students are summatively assessed on their PPVE learning through scenario-based exams (see [14]) and a 3000 word piece of coursework in their third year of the course (see Appendix 1).

Within the PPVE component of the medical curriculum, we introduced a clinical ethics strand that runs throughout the programme (see Appendix 2). Briefly, in the first year, students were introduced to two common ethical frameworks to structure their discussions in small groups whilst they considered cases available through the UKCEN. The focus of the two-hour workshops was on developing students’ decision-making skills.

In the third year, students learn a further two ethical frameworks and are asked to identify a case whilst on placement that they deemed ethically problematic either for themselves and/or the healthcare professionals involved. Students then applied one of the four ethical frameworks they have learned in their first and third years to the case to arrive at a decision as to how to respond or take action to the ethical problem they identified. The purpose of the coursework was to foster students’ ethical awareness and sensitivity, and learn about their professional and legal responsibilities, including safeguarding and whistleblowing. Students were actively encouraged to acknowledge and identify any potential safeguarding and whistleblowing concerns they had in the coursework, as well as with the lead for PPVE. Students were supported when raising any potential safeguarding and whistleblowing matters, which were escalated to the Director of the medical degree and the Director of Education at the Trust so action could be taken if necessary. The coursework also encouraged students to reflect upon the strengths and limitations of using ethical frameworks when making decisions. Being able to critique the frameworks was essential to ensure students remained analytically engaged when working through the case and were encouraged to draw on their wider learning around ethical theories when applying the framework to their case. The students are supported with the coursework in a variety of ways including workshops (see Appendix 3), a short handbook and a printed resource pack (available on request), and an online discussion forum available at set points of the year.

In the fifth year, students engaged in interprofessional clinical ethics forums with Social Work and Clinical Psychology students. They had to present and choose one of their own cases they had identified whilst on placement that they deemed ethically problematic either for themselves and/or the healthcare professionals involved. The case was then discussed within the multiprofessional student group using one of four ethical frameworks. The purpose of the clinical ethics forums was for students to have reflective space to explore cases they had found ethically problematic, whilst also gaining experience of discussing ethical problems in multidisciplinary teams, and hone their decision-making skills for their own and colleagues’ ethical cases. We also aimed for students to recognise that there might often be more than one reasonable course of action to the same problem, as well as acknowledge and respect reasonable differences of opinion.

When designing our clinical ethics activities, our preference for using students’ own cases for the analysis and discussions was informed by the literature. We aspired to bring ethical concepts to life as well as help students understand the moral implications of what they experienced during their training [31]. Similar to Johnston and colleagues [20], we were concerned that unresolved ethical dilemmas encountered by students might erode their ethical principles and ‘ethical self-identities’ and therefore we aimed to provide an outlet for their own cases. We also intended to provide students with practice in moving from the particular situation to general principles and applying what they know about ethics to guide them in their professional and personal lives [31]. In some way, by students using their own cases, it generated an ‘experiential’ curriculum, which aimed to create a sense of comfort with everyday ward ethical issues [41], and give them greater confidence and skill in being alert to, understanding, and sensitively acting upon what they experienced in their medical training and later in practice [31].

We also looked to the literature when deciding to use ethical frameworks, in particular when choosing which frameworks to include and at what point in the students’ training. Ethical frameworks were presented in the literature as providing an action-guide for ethical decision-making in clinical practice, and offered a strategy for clinical ethical reasoning that could be readily taught and grasped by students [32]. Essentially, the frameworks would support students to build their ethical reasoning skills as they approached ethically challenging cases in a systematic fashion within a structured format [41], and therefore helped to manage the ethical aspects of their clinical practice [32].

We wished to show progression in the choice of frameworks, and therefore started with the Four Principles [3] as they are fundamental to all the other frameworks used in later years. New frameworks were introduced in the third and fifth years, such as the Seedhouse Grid [40], that were complex and encouraged students to have in-depth discussions. We chose frameworks that were used in real-world CECs discussions, such as the Four Quadrants [21], and also those frameworks that were created by medical students at other medical schools e.g. C.O.R.E Values [25] to demonstrate relevance of the frameworks to students. We chose frameworks that were accessible to all vocations involved in the interprofessional clinical ethics forums, and were known to be used by qualified practitioners such as C.A.R.E. [39], as we hoped to encourage the translation of ethics knowledge into practice settings [26]. Finally, as a range of facilitators were involved in the clinical ethics activities, including practitioners and academics, we chose frameworks that allowed both clinical- and classroom-based educators to be involved in the teaching of ethics [12].

## Results

Written student feedback was collected during a period of 6 years. Students were asked to provide feedback in response to a series of open questions after submitting the case analysis coursework conducted in the third year of the course, and participating in the interprofessional clinical ethics forums in the fifth year of the course (see Appendix 4). At the time the feedback was collected, the student cohort size was approximately 54 students in each year of the course. Feedback was gathered from all students when they submitted their coursework online, although it was possible for students to write ‘not applicable’ or ‘no comment’ for a question and for submission to still go ahead. It is important to note that whilst not every student answered every question, every student did complete part of the online feedback sheet and therefore provided some feedback. The feedback was shared with all coursework markers, those attending the moderating board, wider department colleagues at learning and teaching committee meetings, and external examiners to explore and identify ways to enhance the design and delivery of the coursework for the following year.

The student feedback was coded thematically [4]. Three higher-level themes emerged that focused upon students’ experience of learning clinical ethics, preparing students for practice, and developing ‘ethical’ practitioners. Within these broad themes, a number of narrower themes emerged that explored students’ decision-making, ethical awareness, and ethical sensitivity in particular.

Whilst we have not conducted pre- and post- testing of students’ decision-making, ethical awareness and ethical sensitivity, we have explored whether students perceived their abilities to have been positively influenced by participating in the clinical ethics teaching and assessment activities. University ethical approval was granted from the Faculty of Health and Medicine Research Ethics Committee.

Written extracts are provided from the student feedback to illustrate points made and the cohort year is provided after each extract. In the feedback extracts, students referred to ‘tools’, which were ethical frameworks such as Four Principles, and Four Quadrants. Students also referred to ‘scenarios’, which are students’ cases from their training that they deemed ethically challenging. As way of context, the coursework in third year was the first time in the course whereby students were required to identify and analyse a case they deemed ethically problematic whilst on placement. Students often struggled with identifying a scenario to discuss within their coursework and required support from facilitators to distinguish a case that had predominately ethical, rather than clinical, uncertainty.

### Learning Clinical Ethics Using Student Cases and Ethical Frameworks

A common theme throughout the feedback for both the clinical ethics forums and the coursework was students’ appreciation of the practical nature of clinical ethics. In particular, students enjoyed the application of frameworks to their cases:

it is a very practical way of learning about ethics. No amount of teaching could replace this experience (2014/2015).

it allowed me to see the practical aspect of things we learned in class (2012/2013)

it allowed me to have real practical experience with engaging with the tool (2014/2015)

For some students, using their own cases to learn clinical ethics highlighted the relevance of the frameworks to practice settings:

I enjoyed applying a clinical tool to an ethical scenario I had experienced myself as I found this more interesting and relevant than using made up scenarios (2014/2015).

The scenario aspect helps in hospital placements because it shows how quickly and easily the tools can be applied in real life (2012/2013)

However, a minority of students implicitly presented a mismatch between what they were taught through the coursework and what they witnessed on the wards. In particular one student queried the relevance of the frameworks in terms of influencing practice in light of the rise of protocols and policies, “I do not think that people analyse scenarios in this nature, it is more guideline orientated in practice, either you can do something or you can’t” (2015/2016).

As students gained experience of applying the ethical frameworks to their own case, the majority of students were able to see the value of the frameworks, especially for clinical practice:

applying the tool to a scenario that I had seen made the tool seem relevant and necessary for clinical practice (2017/2018)

I enjoyed applying the tool to my scenario as it enabled me to learn how I would use it in clinical practice (2017/2018)

I think the ethical tool I used can apply to most ethical situations that arise in clinical setting. Therefore it relates by being of use clinically (2014/2015)

The feedback from the students also suggested that once they were aware of the application and relevance of clinical ethics, it secured students’ future engagement with the frameworks:

It related to my placements as these type of scenarios are experienced regularly. This coursework has allowed me to appreciate how these scenarios could be analysed during placement in the future (15/16)

I feel that I could easily apply my ethical tool on-the-go, having studied it in more depth (15/16)

Although it is worthwhile noting that one student did question the value of learning about the frameworks and the application to real life experiences, “it is so specific to one scenario its difficult to know how the tool will help me in the future” (2013/2014). That said, the majority of students made connections between learning clinical ethics using their own cases during their training and informing their future practices:

Because my scenario was a situation that actually happened to me, it meant I could reflect on how I could act in the future. Which I hope will be useful for my future practice (2014/15)

I found it refreshing to write about ethics and law in context of a real life scenario. I feel I have gained skills about ethics and law to carry forward into clinical practice (2014/2015)

Students also enjoyed their learning being centred on their own ethical case rather than a hypothetical case, and found it enhanced their learning experience:

actually applying the tool to a clinical situation that I’ve experienced. It is much more useful to apply these tools to our own experience rather than a made up scenario (2012/2013)

I also found that analysing a situation that had actually happened to me to be very enlightening (2014/2015)

It was helpful to apply the ethical tool to my own scenario and made it more enjoyable working through a scenario I had been confronted with in clinical practice (2014/2015)

For some students, the application of the framework to their own scenario supported their wider PPVE learning, as it *“gave context to theory” (2012/2013)*. In particular, students referred to their ability to learn and engage with ethics, law and professionalism topics within the wider curriculum,

I enjoyed the applying part of the coursework the most. It helped me to learn certain concepts in detail and also understand ethics better (2017/2018)

helped me understand some new aspects of PPVE which I did not now previously (2014/2015)

Clinical ethics also was presented in the feedback as supporting students’ ability to understand and retain their ethics, law and professionalism learning:

I enjoyed relating PPVE concepts to my personal experience, I feel this allowed me to retain information better (2015/2016)

I liked applying the tool to a real life situation it was a better way to learn and understand it (2015/2016)

Students also claimed that the clinical ethics activities helped to integrate their learning of ethics into their wider clinical learning outside of the classroom, which they seemed to value:

relates to experiences in hospital (2015/2016)

I think that it brings a lot of my learning together rather than it all being compartmentalised (2015/2016)

directly relevant to clinical placements and therefore very useful (2015/2016)

In turn, the relevance of the ethical frameworks to wider clinical practice, was mutually beneficial to the learning of clinical ethics. Some students enjoyed critiquing the ethical frameworks against the needs and demands of clinical practice:

I enjoyed this piece of coursework and think it has made me consider the tools in a more critical way. Rather than just using the tools in my future practise, I will try to look at what is missing from each tool, and how I might need to actually adapt the tool to make it different in different cases (2012/2013)

Applying the ethical tool and further exploring how the tool could be improved by identifying its limitations (2016/2017)

I enjoyed applying the ethical tool the most and critiquing the tool with regard to current practice (2016/2017)

It appeared that students appreciated learning clinical ethics using their own cases and applying ethical frameworks to help structure their analysis of their own cases. Many students valued the practical nature of clinical ethics in terms of applying a framework to support decision-making in the face of ethical uncertainty, and made reference to the relevance of clinical ethics to clinical practice, suggesting that they perceived learning clinical ethics as equipping them for practice.

### Learning Clinical Ethics to be Prepared for Practice

The clinical ethics activities were presented in the student feedback as developing students’ decision-making capabilities around ethical challenges. In particular, students emphasised that the decisions they arrived at in the coursework and the forums were logical and structured:

it helps me to deal with dilemmas in a more logical way (2015/2016)

it just enabled me a systematic way of thinking about decisions for the future (2016/2017)

the ethical tool will help me work through the domains and help me come to a justified decision about a scenario in the future (2016/2017)

Although a minority of students found explaining their reasoning around cases particularly challenging:

explaining reasoning behind choice (2015/2016)

I found it difficult to explain how the tool brought me to the outcome (2015/2016)

In turn, the ethical frameworks meant that students were able to break down a case that was ethically challenging, as well as recognise their emotional responses to cases:

giving me a deeper appreciation of how ethical dilemmas can be broken down and able to come to a more thought through decision (2015/2016)

it made me realise that my own beliefs are powerful when I am judging whether something is right/wrong, and to step back and look carefully at the possible outcomes (2015/2016)

The student feedback on applying an ethical framework to a real-life case suggested that students were reflective whilst making their decision. Furthermore, students were able to reflect upon their own decision-making process. For some students, using an ethical framework highlighted overlooked elements of the case being analysed. Implicit in students’ feedback therefore was the value of using a framework to arrive at an ethically-informed decision:

I enjoyed seeing what different tools are out there and the benefits of a considered decision and the importance of getting ethical decisions right (2013/2014)

applying it to my scenario and reflecting on my own opinion and seeing how there were aspects of the patient care I had missed (2015/2016)

I really enjoyed applying the ethical tool, and seeing how working my way through it helped me to see the situation from different points of view, and actually made me change my original thoughts on what the most acceptable outcome should be (2012/2013)

Students described how their opinions shifted whilst working through the various components of the ethical frameworks, which suggested an openness and flexibility within their decision-making skills:

applying the tools to my scenario as it allowed my opinion to change (2014/2015)

seeing how my own opinions were shaped as I did it (2012/2013)

it was useful to work through my particular scenario, and see how it can help you to change your mind (2012/2013)

Equally, the clinical ethics activities provided students with the opportunity to examine others’ decision-making when faced with the same case:

it has also enhanced my understanding of ethical decision making in healthcare” (2012/2013)

considering why a doctor might make a certain choice and what might be reasons not to make those choices. Allowed me to look at clinical decision making in more detail (2017/2018)

made me realise how open ethical situation is to being interpreted differently (2013/2014)

Clinical ethics was presented by students as supporting students’ decision-making skills in the future by providing them with a ‘tool kit’ that they could draw on in clinical practice:

it has shown me that when I may come across tricky ethical situations in practice, that tools are available to help analyse what to do (2015/2016)

allows for use of a tool which is now familiar to be deployed in real world settings (2014/2015)

it has allowed me to think about how to approach any ethical decisions I face in the future, whether that be in the hospital or the community (2014/2015)

The notion of clinical ethics as equipping students for practice and providing a ‘tool kit’ was also reflected in how students’ chose which ethical framework to use in their coursework. Students emphasised the clinical relevance of some ethical frameworks and how applicable the frameworks would be for future practice:

I chose the tool I found most interesting and most clinically relevant to me when I am a doctor (2012/2013)

I wanted to choose an ethical tool that I would use in the future – so easy to remember and relevant to all scenarios (2014/2015)

I also wanted to use a tool that I would easily be able to use in practice, so I chose one that was easy to remember (2013/2014)

The clinical ethics activities compelled students to make a decision, which demonstrated to them progression in their learning, whilst also generating an opportunity to integrate their wider learning:

having to think critically about an ethical decision and actually make a decision rather than simply listing pros and cons of each outcome (2015/2016)

I found that coming up with a plan of action based on codes of practice and regulations was the most enlightening part of the coursework (2014/2015)

However, the practical nature and focus of the coursework meant for some students that the written component was a limitation and unnecessary:

I think that it would be useful to do 10 min presentations instead…I feel that ethics is better spoken about and discussed…a 10 min presentations in front of a panel would have still taught me what I needed to know without spending hours on trying to write about them (2015/2016)

Within the student feedback, clinical ethics was presented as supporting the development of ethical practitioners. Students perceived clinical ethics as providing a tool kit that they could draw on when qualified and on the wards. Students were reflective in and around their decision-making, and demonstrated sensitivity towards others’ perspectives, and an awareness of the importance of making ethical decisions. Consequently, it was inferred from the feedback that students perceived learning clinical ethics as helping them to become ethical practitioners.

### Learning Clinical Ethics to Become ‘Ethical’ Practitioners

The majority of students described how their ethical awareness and sensitivity were enhanced as a result of engaging in the clinical ethics activities. Students also made connections between their learning of clinical ethics with their current and future healthcare practice being positively influenced.

#### Ethical Awareness

Students reported being more aware of the ethical aspects of cases that were discussed and analysed in the clinical ethics activities. They also claimed to be able to identify ethical aspects of cases as a result of using the ethical frameworks:

I enjoyed applying the tool to my scenario and seeing different things which I wouldn’t have normally seen (2013/2014)

applying the ethical tools to my case was interesting as it taught me more about all aspects of the case (2017/2018)

I enjoyed applying the tool to my own scenario and reflecting on aspects of the dilemma that I had not considered previously (2014/2015)

To some extent, the feedback suggested that students had developed an expanded view of their cases, which incorporated other people’s perspectives that they would not previously had considered. In some extracts, it can be inferred that engaging in clinical ethics had shifted and challenged students’ usual way of thinking about clinical cases:

I enjoyed applying the ethical tools the most as it made me think in a way I normally wouldn’t (2014/2015)

It highlighted to me the need to think of the wider considerations when trying to make an ethical decision (2013/2014)

it made me consider dilemmas that I had never thought of before. It was an insightful learning experience (2012/2013)

The clinical ethics activities required students to draw on their own ethical dilemmas, and in turn had fostered some students to develop an ‘ethical lens’ whilst on clinical placements. The feedback suggested that students had gained insight into the ethical components of their clinical placements, and the clinical ethics activities had encouraged them to engage with the placements in a different way:

opens your eyes to all ethical dilemmas that you may not have seen before (2015/2016)

It has made me more aware of ethical scenarios that I face in hospital (2014/2015)

allowed me to put my hospital experience into context and consider ethical dilemmas which might sometimes be overlooked (2013/2014)

The emphasis of the clinical ethics activities on developing students’ ethical awareness and fostering an ‘ethical lens’ to their clinical placements was also reflected in the feedback when students were asked what they found most challenging during the clinical ethics activities. Some students reported struggling to identify a case for discussion that they considered had an ethical dimension:

I found it challenging to think of a scenario. I felt like I hadn’t seen many ethical situations in clinical practice (2015/2016)

finding a scenario that was a genuine ethical dilemma. Rarely does something morally questionable happen (2013/2014)

I felt that it was quite hard to find an ethical dilemma as I feel I didn’t come across many (2012/2013)

When examining the feedback closer to understand this challenge for the students, it appeared that some students perceived medical specialities as providing more or less ethical cases:

finding a scenario since it took a while. I think it depended on which rotations you had seen so far (2015/2016)

I found finding a scenario very stressful and difficult given the rotations I had done (2015/2016)

In addition to this, these same students tended to ‘overlook’ and were dismissive of the ‘everyday ethics’ challenges that permeate medical practice. This was confirmed when students attended placements in the same medical specialities and emphasised that the clinical ethics activities had given them an appreciation of the ethical aspects of everyday practice:

very applicable, we encounter similar issues on the wards and in clinics. The dilemmas are not always big but they are important for those involved with the care and the patient (2012/2013)

very relevant to everyday practice, particularly in GP setting (2012/2013)

has given me more of an appreciation of how ethics comes into all clinical decision making and all those aspects need to be considered in all environments (2013/2014)

#### Ethical Reflection

The clinical ethics activities also appeared to promote and prompt reflection by the students on their clinical experiences. For some students, the activities provided space and time for them to reflect on the ethical aspects of their experiences during hospital and community placements:

allows me to think about ethical issues that arise in hospital routinely and allows me to reflect on this (2015/2016)

I enjoyed the opportunity to stop and reflect in detail on a clinical experience (2014/2015)

helpful in terms of thinking about ethical scenarios that I have encountered in hospital etc. Often don’t necessarily take the time to think about them and about how they could be resolved in the future (2015/2016)

Students also suggested that the clinical ethics activities prompted them to reflect on their own and others’ practices whilst learning:

The coursework made me think about the ethical consequences of my actions on placement (2012/2013)

it makes me consider the ethics of everyday things a lot more and wonder if some of the situations you see on the wards were analysed using an ethical tool would the outcome be the same as the ones that are taken clinically (2012/2013)

Some students also made the association between the opportunities to reflect on ethical aspects of their wider learning during placements with their future practices,

it helps to improve upon my reflection skills for future hospital practice (2014/2015)

It will help [me] think in a more ethically critical way when approaching situations in hospital (2012/2013)

I will try to reflect more on my observations in hospital, community e.g. was a consultant’s clinical decision/behaviour appropriate in a certain situation (2012/2013)

#### Ethical Practices

The student feedback highlighted that students perceived learning about clinical ethics as having the potential to inform their current and future clinical practice. Students described how useful having knowledge and experience of applying a range of ethical frameworks was whilst on clinical placements. Consequently, clinical ethics was presented as providing ‘scaffolding’ to support students during placements, and in turn the relevance of clinical ethics was confirmed:

Helps you consider how you would handle ethical scenarios whilst on placement around the hospital (2015/2016)

I will try to apply the ethical tool to other histories I take in hospital (2014/2015)

Feels mainly applicable to clinical work. Stimulates thought-processes whilst on placements and helps students to question proceedings (2014/2015)

For some students, they viewed the ethical frameworks as being able to assist them in their future practices, in particular their decision-making:

I think looking at ethical situations that are commonplace in clinical settings can go a long way to informing my practice and ethical decision making further down my career (2014/2015)

I hope it will assist me in making ethical decisions in the future (2015/2016)

It will help me address better future ethical dilemmas in the hospital setting (2012/2013)

Whilst others referred to how learning about clinical ethics would support their general healthcare practices, including working as part of a multidisciplinary team:

Having to read about the ethical tools and their applications has been incredibly helpful especially in terms of understanding how we can use them in daily practice (2012/2013)

the session helped to interact with other healthcare professionals which I think is particularly useful for when I become a doctor and will have to work as part of a team with multiple members of staff (2017/2018)

The interdisciplinary discussion was both rich, thoughtful and rewarding and helped break down some barriers (2016/2017)

Some students also considered their engagement in the clinical ethics activities had assisted their development as an ‘ethical’ practitioner:

Helps to consider my personal ethical perspective and how I aim to be once I begin practise (2015/2016)

it helps in shaping me as a better future doctor overall (2014/2015)

Within the student feedback, it was apparent that students recognised the benefits of learning through and engaging in clinical ethics activities, and acknowledged the rewards from this learning and engagement to them as medical students and as tomorrow’s doctors.

## Discussion

In this paper, we have examined student feedback from engaging in a range of clinical ethics activities during the third and fifth years of an undergraduate medical degree. Our findings confirmed claims made by others whereby students integrate their wider learning when engaging in clinical ethics activities. Clinical ethics brings the ethical theory to life for students [31] and helps them develop and experience decision-making when faced with ethical uncertainty [1, 32]. Throughout the feedback, the benefits to receiving training in clinical ethics were apparent to medical students to support their learning during clinical placements, whereby the ethical frameworks acted as scaffolding and provided a ‘tool kit’ to be drawn upon as others have proposed [32, 41]. Similar to other clinical ethics educators, our students appreciated the opportunity to reflect on the ethical aspects of their clinical placements when engaging in the clinical ethics activities [20, 37]. The students also anticipated how learning about clinical ethics during their medical degree would benefit them in their future clinical careers as they had been given the opportunity to develop their decision-making skills, and appreciated the value of discussing ethical matters with a multidisciplinary team. We infer from these statements that by our students engaging in clinical ethics activities we have encouraged the transmission of ethics knowledge into practice settings, although we do accept that our results are reporting students’ thoughts, attitudes and beliefs rather than observed behaviour [26]. Our findings challenge those who question value, purpose, and role of ethics training [17, 28]. Instead, our research suggests that clinical ethics teaching at undergraduate level can have a positive influence on tomorrow’s doctors development and help to foster ethical practitioners of the future [12, 31].

There are wider benefits arising from introducing tomorrow’s doctors to clinical ethics. Arguably, educators are securing the future of clinical ethics support. For example, students’ awareness of the value of CECs through such clinical ethics activities described in our paper may mean they will consider joining a CEC in the future when they qualify, or look to establish a CEC in a Trust where one does not exist. Given that clinical ethics support has historically functioned as a voluntary system, then providing clinical ethics training to tomorrow’s doctors creates the next generation of potential ‘volunteers’ [1, 46], and prevents the idea of clinical ethics support being entirely phased out in the NHS [2]. Whether the basis of clinical ethics support in the UK should be (solely) reliant on goodwill however when healthcare organisations and patients, benefit is a question that remains to be answered.

For an organisation then, there are an array of benefits by providing students with the opportunities to engage in clinical ethics activities, such as addressing the ethics training of and availability to current practitioners [9, 15, 23]. Arguably, current practitioners have the opportunity to learn through medical students and junior doctors whilst on clinical placement if ethical aspects of cases are raised during team meetings and they are involved in the discussions. Furthermore, current practitioners also have the opportunity to learn about clinical ethics vicariously if they are involved in clinical ethics activities, such as facilitating clinical ethics forums and marking the coursework. As an organisation itself, the current and future practitioners are likely to be ethically sensitive and aware, with enhanced decision-making skills, and a tool kit to draw upon when facing ethical cases in practice. They are therefore able to inform practices and influence the culture within the NHS [26]. They are also likely to appreciate the value and importance of gaining insight into multiple perspectives when discussing ethical aspects of cases with a range of professionals. Having such skills therefore are not to be underestimated given that the majority of practitioners in the UK are having to fend for themselves when facing ethical dilemmas with the dwindling number of registered CECs [2].

However, there is a danger that tomorrow’s doctors are being made aware of the value of multidisciplinary discussions through clinical ethics activities, and yet when qualified no such outlet such as CECs exist within their Trust. Furthermore, in order to facilitate a cross-pollination of learning of clinical ethics between current and tomorrow’s practitioners a willingness and an open mind is required on behalf of the current practitioners [Talash et al., accepted]. There needs to be honesty surrounding how challenging senior practitioners can find dealing with their own and junior colleagues’ ethical dilemmas, and how heavy the felt burden is in order to have the ‘answers’ to the dilemmas they face in practice [Machin & Wilkinson, accepted]. The perception of ethics training also needs to evolve so that it is acknowledged that ethics learning is never ‘done’, and instead is required just as much after medical school as during [23]. In turn, being able to learn through medical students, engage in clinical ethics training, as well as having access to CECs does go some way to address the ongoing and lifelong nature of learning about ethics [34].

Our findings add weight to those who highlight the significant role that the organisation plays in developing and fostering ethical practice and ethical practitioners [19, 42, Talash et al., accepted]. It is evident from our findings that partnerships need to be formed between medical schools and hospital Trusts, particularly around the continuation of students’ learning of ethics. Our students reported that the ‘everyday ethics’ tended to be overlooked. Moreover, some students perceived some medical specialities to have more or less ethical challenges. It is possible that this perspective had been generated through current practitioners’ attitudes, behaviours and actions. Our findings therefore support the significant influence that role modelling plays in the development of tomorrow’s doctors [7, 34]. We therefore need those working at the coalface to pick up the baton initiated at medical schools to support, foster, and continue the clinical ethics learning. In some way, the student feedback highlighted that ethics is just as much about what you do when someone *is* watching, as not watching. It is therefore vital that the importance of the commonplace, recurring and frequent ethical aspects of healthcare are pointed out to medical students during their training in order for them to appreciate the significance of them, as well as be prepared for practice.

Equally, it is possible that such perceptions of everyday ethics had been absorbed through the organisational culture that current and tomorrow’s practitioners were operating within. After all, if there is a lack of acknowledgement of the everyday uncertainty that practitioners grapple with, as demonstrated through the lack of investment in training and clinical ethics support for community and hospital based practitioners [Machin & Wilkinson, accepted], then it is hardly surprising that students adopt the view that the everyday ethics do not matter. From our study, it is apparent that the ‘hidden curriculum’ [16] extends beyond the individual practitioner to the organisation itself, and suggests that ethical leadership needs to be a priority within the NHS. As others have argued elsewhere [19, 42], it is vital we develop a culture within the NHS where the everyday ethics matters and the burden that practitioners carry when delivering care is demonstrated through investment from senior leaders in developing clinical ethics support at a local, regional and national level.

## Data Availability

Not applicable.

## Appendix 1: A Brief Overview of the Structure for the Case Analysis Coursework for Third Year Medical Students

1. Scenario (up to 500 words)
  Choose one experience based on your current rotations. It is likely to be an experience that raises professional, moral or ethical dilemmas for the healthcare professionals involved. Describe people’s involvement, actions, statements, body language during the experience. Also, make explicit the legal, ethical and professional context of the experience.
2. Briefly describe ethical framework (up to 500 words)
  Choose an ethical framework that you have come across during your training so far, such as Four Principles plus Scope, Four Quadrants, Seedhouse Grid, and CoRE Framework, and explain it so that a lay person could replicate applying the framework to another scenario.
3. Apply ethical framework (minimum of 500 words)
  In a logical and concise way, apply the tool to your experience. Make clear the stages of the framework you are referring to. You may wish to use sub-headings for each stage. The application of the ethical framework to your scenario will not be value-free, therefore you need to be explicit about what assumptions and beliefs you might be basing the analysis on.
4. Outcome (up to 500 words)
  The outcome is the decision and/or plan of action that you would take in the scenario if you followed the ethical framework.
5. Reflection on the Outcome (minimum of 500 words)
  Reflect on the outcome you came to by using the ethical framework. Do you agree or disagree with the outcome? Is the outcome permissible in practice according to professional or legal guidance? What would the outcome mean for the people involved in the scenario?
6. Critique of the ethical framework (minimum of 500 words)
  Reflect on using the ethical framework to a real-life case. What are the limitations and strengths of the framework? How did you find the process of applying an ethical framework as a way of achieving guidance for a real-life case? Would you use the framework again in future? Would it work in all environments—on the ward, in the surgery? Would it be appropriate to use with clinical team members?

## Appendix 2: A Brief Overview of the Clinical Ethics Strand Introduced to Lancaster Medical School

Throughout the Clinical Ethics strand small group work is essential. It conveys the message to the students that ethics is a practical skill, and requires that difficult cases be discussed and debated within teams. It also has the added benefit of developing students’ abilities to give and receive challenge and argument in a constructive and respectful manner. Students are introduced to ‘paper patients’ using cases from UK CEN in the first couple of years of their training in order to introduce concepts in a non-threatening manner. Students then move onto cases based on their own experiences or observations, which is needed to raise their sensitivity to recognising ethical dilemmas, while also bringing their wider learning of ethics, law and professionalism to life.

**Table:**

Year 1: Decision making workshops	Year 3: Case analysis	Year 5: Clinical ethics forums
Medical students only Introduced to clinical ethics (brief history, current state in UK, what it is, purpose, UK CEN, Committees) Introduce ethical frameworks (Four Principles and Four Quadrants), provided with scenarios (from UK CEN), worked through in pairs, with clinical and academic facilitators Maps on to wider learning (Four Principles and Four Quadrant as PBL learning objectives and recommended resources, and lectures)	Medical students only Case Analysis Workshop: Four Principles and Four Quadrants Case Analysis Workshop: Seedhouse Grid and Core Values Compass/Grid Online Discussion Forum open at set times of the year to allow students to pose questions relating to the coursework and for facilitators to respond Case Analysis Coursework	Medicine, (Masters) Social work, and (Doctoral) Clinical Psychology students Share cases witnessed during placements Choose a case and choose a framework (Four Principles and Four Quadrants, Seedhouse Grid, and C.A.R.E) Discuss the case/s using the framework/ Offer guidance to student presenting the case Reflect on the case, using the frameworks, participating in MDT discussion around ethical cases Supported by practitioner and academic facilitators

## Appendix 3: Outline of First Case Analysis Workshop for Third Year Medical Students

### Set up

- Two facilitators, one clinical and one academic
- Large flat room
- Approx 2 h and a half hours in length
- PowerPoint slides available on request

### Content

Introduction (approx. 15 min)

- Purpose of workshop, structure of workshop, norms for the day
- Introducing the coursework (purpose, format, practicalities)
- Previous students’ views on coursework
- Guidance on how to choose scenario
- Tips from previous students on how to choose an ethical framework

First small group exercise (approx. 40 min)

- A reminder of the Four Principles.
- Read previous student scenario, apply Four Principles, decide in group what ‘guidance’ could be formulated for the scenario based on framework, and be prepared to explain your decision and rationale.
- Ask students to change groups and discuss how you applied the framework to the scenario, what took priority and why, what information do you need, what questions do you need answering before proceeding.

Large group discussion (approx. 20 min)

- Do you agree or disagree with the outcome arrived at by using the framework? Is the outcome permissible in practice according to professional or legal guidance? What would the outcome mean for the people involved in the scenario?
- Feedback from clinical facilitator on the clinical components of the scenario, and questions raised by students during discussion points.
- Guidance from academic facilitator relating to completing the coursework and responding to questions from students during discussion points.

Break (approx. 10 min)

Second small group exercise (approx. 30 min)

- A reminder of the Four Quadrants.
- Re-read the previous student scenario, and apply Four Quadrants, decide in group what ‘guidance’ could be formulated for the scenario based on framework, and be prepared to explain your decision and rationale.
- Ask students to change groups and discuss how you applied the framework to the scenario, what took priority and why, what information do you need, what questions do you need answering before proceeding.

Large group discussion (approx. 15 min)

- How did you find the process of applying the Four Principles and the Four Quadrants as a way of achieving guidance for a case?
- What are the limitations and strengths of using the Four Principles and Four Quadrants?
- Would you use the Four Principles and Four Quadrants again in the future?
- Would they work in all environments—on the ward? In the GP surgery? A&E, ICU?
- Would they be appropriate to use with clinical team members?

Close (approx. 10 min)

- Final thoughts from students and facilitators
- Tips from previous students on writing the coursework

## Appendix 4: A Selection of Student Feedback Questions for Clinical Ethics Sessions

Some Case Analysis Coursework Feedback Questions

- What elements of the coursework did you enjoy most e.g. learning about the ethical frameworks, applying the ethical frameworks to your own case?
- How does the content of your coursework relate to your wider learning elsewhere i.e. hospital, community, PBL, lectures, SSMs?
- How did you choose the ethical framework to apply to your own case?
- What elements of the coursework did you find most challenging e.g. learning about the ethical frameworks, applying the ethical frameworks to your own case?

Some Interprofessional Clinical Ethics Forums Reflection Prompts

- In what way has the session challenged or changed your attitude to professionalism and working in interdisciplinary teams?
- What have you learned about challenging the behaviour of professional colleagues?
- What skills have you learned about working through legal or ethical problems in a multidisciplinary team?
